# Altered early immune response after fracture and traumatic brain injury

**DOI:** 10.3389/fimmu.2023.1074207

**Published:** 2023-01-25

**Authors:** Melanie Haffner-Luntzer, Birte Weber, Kazuhito Morioka, Ina Lackner, Verena Fischer, Chelsea Bahney, Anita Ignatius, Miriam Kalbitz, Ralph Marcucio, Theodore Miclau

**Affiliations:** ^1^ Institute of Orthopaedic Research and Biomechanics, University Medical Center Ulm, Ulm, Germany; ^2^ Department of Orthopaedic Surgery, Orthopaedic Trauma Institute, University of California, San Francisco, San Francisco, CA, United States; ^3^ Department of Traumatology, Hand-, Plastic- and Reconstructive Surgery, University Medical Center Ulm, Ulm, Germany; ^4^ Department of Trauma−, Hand− and Reconstructive Surgery, University Hospital Frankfurt, Goethe-University, Frankfurt am Main, Germany; ^5^ Department of Neurological Surgery, Weill Institute for Neurosciences, Brain and Spinal Injury Center (BASIC), University of California, San Francisco (UCSF), San Francisco, CA, United States; ^6^ Steadman Phillipon Research Institute, Vail, CO, United States

**Keywords:** fracture healing, traumatic brain injury, inflammation, mast cells, polytrauma

## Abstract

**Introduction:**

Clinical and preclinical data suggest accelerated bone fracture healing in subjects with an additional traumatic brain injury (TBI). Mechanistically, altered metabolism and neuro-endocrine regulations have been shown to influence bone formation after combined fracture and TBI, thereby increasing the bone content in the fracture callus. However, the early inflammatory response towards fracture and TBI has not been investigated in detail so far. This is of great importance, since the early inflammatory phase of fracture healing is known to be essential for the initiation of downstream regenerative processes for adequate fracture repair.

**Methods:**

Therefore, we analyzed systemic and local inflammatory mediators and immune cells in mice which were exposed to fracture only or fracture + TBI 6h and 24h after injury.

**Results:**

We found a dysregulated systemic immune response and significantly fewer neutrophils and mast cells locally in the fracture hematoma. Further, local CXCL10 expression was significantly decreased in the animals with combined trauma, which correlated significantly with the reduced mast cell numbers.

**Discussion:**

Since mast cells and mast cell-derived CXCL10 have been shown to increase osteoclastogenesis, the reduced mast cell numbers might contribute to higher bone content in the fracture callus of fracture + TBI mice due to decreased callus remodeling.

## 1 Introduction

Despite the remarkably high regeneration capacity of the skeletal system as well as ongoing improvement in fracture treatment during recent decades, orthopaedic complications such as delayed fracture healing or non-unions are still challenging (1). The healing process of bone is strongly dependent on age, trauma severity, fracture fixation, existing comorbidities and other biomechanical and biological factors (2, 3). It has been shown that severe trauma might be a risk factor for orthopaedic complications, especially an additional thoracic trauma or hemorrhagic shock was demonstrated in preclinical models to delay bone regeneration (4–7). On the other hand, both clinical and pre-clinical data suggest that an additional traumatic brain injury (TBI) might lead to accelerated fracture (Fx) healing (8–10), although clinical data are not consistent (11). Bigger fracture calli with higher bone content were found in patients and animals with combined Fx and TBI. Furthermore, TBI patients are more prone to heterotopic ossification (12). Preclinical studies investigating the molecular mechanisms behind this phenomenon linked the additional traumatic brain injury to alterations in metabolism and neuro-endocrine regulations (13, 14). Further, inflammatory mediators were altered in the intermediate phase of fracture healing (14). However, the very early systemic and local inflammatory response towards Fx+TBI has not been investigated in detail so far. This is of great importance in this context, since the early inflammatory phase of fracture healing is known to be essential for the initiation of downstream processes for adequate fracture and tissue repair (15–17). Disturbances in this highly complex process consequently result in delayed or impaired healing, as for example demonstrated by the surgical removal of the fracture hematoma (18, 19). In contrast, an overwhelming local inflammation, induced by immune cell activating agents or systemic immune responses in polytrauma patients also disturbs bone regeneration (19, 20). Among the immune cells present in the hematoma, mast cells and polymorphonuclear neutrophils dominate early after fracture with their non-specific defense mechanisms (21–23). MC-mediated neutrophil recruitment has been shown during fracture healing (24–26) and is also reported in chronic inflammatory diseases (27–29). Therein, MCs regulate vascular leakage and attract neutrophils *via* IL-1β, TNF, KC, and MIP-2 (30–33). Downstream, neutrophils recruit macrophages to the fracture site, which have been shown to be of utmost importance for bone regeneration (34). Besides innate immune cells, also cell populations of the adaptive immune system were found to be involved in fracture healing (35) (36, 37). Especially mast cells (MCs) were shown to be master regulators during the early inflammatory phase of bone regeneration, as they appear during the whole time course of fracture healing, interacting with both innate and adaptive immune cells (24, 25, 38–40). Effector T cells are attracted by MC-derived RANTES and antigen presentation of MCs to cytotoxic T cells was shown (41). Various MC-derived chemokines and leukotrienes additionally contribute to T cell recruitment in distinct inflammatory scenarios (39, 41, 42). Therefore, the aim of this study was to analyze the presence of inflammatory mediators and various immune cells in the circulation and locally in the fracture hematoma early after Fx or combined trauma (Fx+TBI). These data should give additional insights into molecular mechanisms which might be responsible for accelerated fracture healing in case of additional head trauma.

## 2 Methods

### 2.1 Experimental design

24 male C57BL/6J mice (provided by Jackson Laboratories) were included in the present study at the age of 10-12 weeks and a body weight of 25-30 g. All experiments were approved by the local animal welfare committee (IACUC UCSF AN143402-03B) and were performed in compliance with international regulations for laboratory animal welfare and handling (ARRIVE guidelines for animal experiments). Half of the mice received a unilateral tibia fracture, and the other half received an unilateral tibia fracture and an ipsilateral traumatic brain injury. 6 mice per group were euthanized at 6h after injury and 24h after injury, respectively. Blood was collected and tibiae were embedded into paraffin for further analysis.

### 2.2 Tibia fracture

Mice anaesthesized with 2% isoflurane were placed in a pronated position under a fracture apparatus. The apparatus consists of a blunt two-pronged base to frame the tibia and a 2 mm-thick blunt punch connected to a guided 500 g weight. The right tibia was centered in the frame under the punch before the weight was lifted to 5 cm above the tibia. When dropping the weight, a closed fracture was created *via* three-point bending. The fracture was not stabilized, and the animals were allowed to move freely after the surgery. The animals received pain medication by buprenorphine injections (sustained-release buprenorphine HCl 1.2 mg/kg) every 6 h. Fracture location and full fracture were confirmed intraoperatively by radiological examination with a Fluoroscan device.

### 2.3 Traumatic brain injury (TBI)

Ipsilateral traumatic brain injury was conducted as described previously (14). Briefly, controlled open cortical contusions were applied on the left side of the brain by compressing the cortex 1.7 mm at a rate of 4.5 m/s for 150 ms using a 3 mm wide convex probe. After contusion, the cortex was covered with saline-soaked gelfoam and the wound was closed in separate anatomical layers using sterile sutures. Animals were closely monitored after the injury. Mice received a peri-operative dose of sustained-release buprenorphine HCl (1.2 mg/kg) as an analgesic.

### 2.4 Sample collection

Mice were euthanized using carbon dioxide. Blood was taken by cardiac puncture. Plasma was collected after centrifugation for 5 min (800 x g, 4°C) and a second centrifugation step for 2 min (13000 x g, 4°C). The samples were stored at -80°C until further analysis. Fractured tibiae were removed, fixed in 4% formalin for 48h, decalcified for 14 days by EDTA and embedded into paraffin.

### 2.5 Multiplex analysis

To analyze systemic inflammatory mediators, plasma from mice was analyzed by using the ProcartaPlex Immunoassay (ThermoFisher, Waltham, MA, USA) for granulocyte-colony stimulating factor (G-CSF), interleukin (IL)-6, keratinocyte chemoattractant (KC), IL-10, tumor necrosis factor (TNF), CXCL10 and monocyte chemoattractant protein-1 (MCP-1). All procedures were performed according to the manufacturer’s instructions. Some plasma parameters have been published as control samples in a previous study regarding cardiac inflammation after trauma (43).

### 2.6 Histology, immunohistochemistry, immunofluorescence

Paraffin-embedded tibiae were cut for histological analysis, immunohistochemistry and RNA analysis from formalin-fixed, paraffin-embedded (FFPE) sections. First, tibiae were cut to 7 µm thick longitudinal sections for histological and immunohistochemical analysis. Toluidin blue staining was conducted to analyze mast cell numbers, as granula of mast cells appear as dark violet in this staining. Afterwards, two 15 µm thick RNase-free sections from each block were cut serially and stored in RNase-free tubes at -20°C until further processing. Before cutting, the blade of the microtome and all other used materials were treated with RNaseZap to avoid RNase contaminations. As described below, RNA can be isolated from FFPE sections by using a specific RNA isolation kit. This technique allowed us to use all mice simultaneously for histological analysis, immunohistochemical staining and qPCR analysis. The fractured bones were cut until the bone marrow was visible on both sides of the fracture making sure that the middle part of the fracture hematoma was displayed on the slices. With this technique, we made sure that always the same area was analyzed.

Staining for Ly6G, F4/80, CD8 and CXCL10 was performed using the following primary antibodies incubated overnight at 4°C: rat anti-mouse Ly6G (1:200; 127632, BioLegend, San Diego, CA, USA) and rat anti-mouse F4/80 (1:500; #MCA497GA, Biorad, Hercules, CA, USA), goat anti-mouse CXCL10 (1:50; #AF-466-NA, R&D systems), rabbit anti-mouse CD8 (1:500, Bioss #bs-0648R). As secondary antibodies, goat-anti rabbit IgG-biotin (1:200; #B2770, Life Technologies, Carlsbad, CA, USA) and goat anti-rat IgG-biotin (1:100 and 1:200 respectively for Ly6G and F4/80 staining; A10517, Invitrogen, Carlsbad, CA, USA) were used and incubated at room temperature (RT) for 30 min or 1 h, respectively. For signal detection, horseradish peroxidase (HRP)-conjugated streptavidin (#PK-6100, VECTASTAIN^®^ Elite ABC-HRP Kit, Peroxidase, Vector Laboratories, Burlingame, UK) was applied according to the manufacturer`s protocols. NovaRED (#SK-4800, Vector^®^ NovaRED^®^ Substrate Kit, Peroxidase (HRP), Vector laboratories) was used as chromogen and the sections were counterstained with hematoxylin (1:2000; #2C-306, Waldeck, Münster, Germany).

Immunofluorescence double staining for CXCL10 and Avidin was performed using the following antibodies: goat anti-mouse CXCL10 (1:50; #AF-466-NA, R&D systems) and Avidin Texas Red (1:150 A820, ThermoFisher) incubated at RT for 1 h. Rabbit anti-goat IgG (H+L) FITC (#A16143, Life Technologies) was used in a concentration of 1:50 for CXCL10 staining as the secondary antibody. Species-specific non-targeting immunoglobulins were used as isotype controls. We have demonstrated previously that Avidin is a very good tool to stain mast cells in tissue sections in various animal models (24, 25, 44, 45).

### 2.7 RNA isolation and qPCR

Total RNA isolation was performed using the FFPE RNEasy kit from Qiagen and RT-PCR was performed as described previously (46). Quantitative RT-PCR was performed using the SensiFAST SYBR Hi-ROX One-Step Kit (Bioline, Memphis, TN, USA). *B2m* was used as the housekeeping gene (F: 5′-ccc gcc tca cat tga aat cc-3′, R: 5′-tgc tta act ctg cag gcg tat-3′). Relative gene expression of TNFa (5’- GGC CAC CAC GCT CTT CTG TCT ACT -3’, 5’- TGA TCT GAG TGT GAG GGT CTG GGC -3’), IL1beta (5’-aca agg aga acc aag caa cg-3’, 5’-ggg tgt gcc gtc ttt cat ta-3’), IL-6 (5’-tcc ttc cta ccc caa ttt cc-3’, 5’-gcc act cct tct gtg act cc-3’), IL-10 (5’-GGC AGA GAA GCA TGG CCC AGA AAT C-3’, 5’-ACT CTT CAC CTG CTC CAC TGC CT-3’) and CXCL10 (5’-GGATCCCTCTCGCAAGGA-3’, 5’-ATCGTGGCAATGATCTCAACA-3’) was calculated using the delta-delta CT method (relative to B2m and the Fx group).

### 2.8 Statistical analysis

Group size was n=6 for each treatment and time point. Data from Fx and Fx + TBI groups were compared by using the unpaired Student’s t-test. P-values of less than 0.05 were considered as statistically significant. Correlation analysis was done by matching CXCL10 protein expression scores from each mouse to the cell counts from the same mouse at both 6h and 24h. Data were analyzed by simple linear regression. Statistical analysis and graphs were done by GraphPad Prism 9. Data are displayed as mean + standard deviation with individual values indicated as black dots for the Fx group and black boxes for the Fx+TBI group.

## 3 Results

### 3.1 Systemic inflammation after fracture and TBI

To analyze systemic inflammation after fracture and combined trauma, several pro- and anti-inflammatory mediators known to be involved in fracture healing were determined in plasma samples at 6h and 24h after injury (**Table 1**). G-CSF, IL-6 and IL-10 levels did not differ between Fx and Fx+TBI mice at all time points. KC was significantly increased in the combined trauma group at 6h, but not at 24h after injury. MCP1 was significantly increased in the combined trauma group at 24h, but not at 6h after injury. CXCL10 was significantly reduced in the Fx+TBI mice at both time points. These data indicate a dysregulated systemic inflammatory response after combined trauma. In general, variations of cytokine levels between the individual mice of one group were especially high in the Fx+TBI group 6h after trauma, which might be due to the combination of two traumata and could influence conclusions draws from that data.

**Table 1:** Inflammatory mediator levels in the plasma.

	6h		24h	
	Fx	Fx + TBI	Fx	Fx + TBI
Plasma				
** G-CSF**	40.7 ± 12.3	154.6 ± 172.4	84.4 ± 93.8	94.1 ± 12.2
** KC**	253.7 ± 131.1	483.8 ± 129.9*	107.5 ± 102.5	91.5 ± 7.9
** IL-6**	155.6 ± 56.8	291.7 ± 281.4	207.7 ± 260.3	120.6 ± 68.0
** IL-10**	6.2 ± 6.9	13.6 ± 4.1	16.9 ± 14.2	29.6 ± 12.1
** CXCL10**	87.2 ± 35.5	40.5 ± 29.7*	129.1 ± 48.1	66.7 ± 12.1*
** MCP1**	30.2 ± 7.9	50.41 ± 20.5	49.9 ± 13.8	68.9 ± 12.0*

*Significantly different (p<0.05) compared to the Fx group, Student’s t-test, Fx, isolated fracture; Fx + TBI, fracture and additional traumatic brain injury; IL, Interleukin; CXCL, C-X-C motif chemokine ligand.

### 3.2 Local expression of pro- and anti-inflammatory mediators in the fracture hematoma

**Figure 1:**
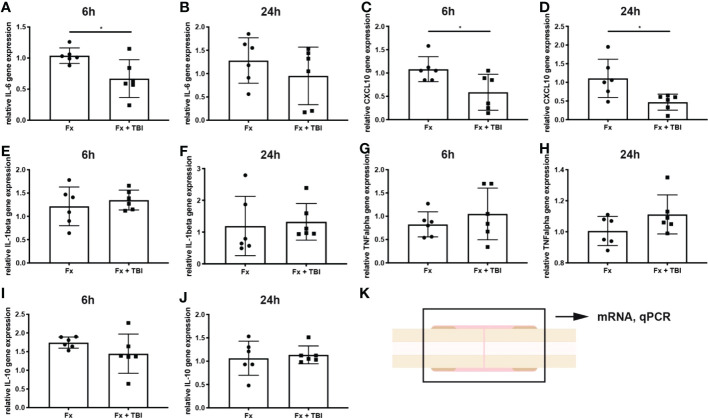
Local gene expression of inflammatory mediators in the hematoma. RNase-free sections of the fractured bones were cut, RNA was isolated and gene expression analyzed by qPCR. Data are normalized to the housekeeping gene B2M and the Fx group, respectively. **(A)** Relative IL-6 gene expression at 6h and **(B)** 24h after injury. **(C)** Relative CXCL10 gene expression at 6h and **(D)** 24h after injury. **(E)** Relative IL-1beta gene expression at 6h and **(F)** 24h after injury. **(G)** Relative TNFalpha gene expression at 6h and **(H)** 24h after injury. **(I)** Relative IL-10 gene expression at 6h and **(J)** 24h after injury. **(K)** Experimental design. * indicates p-value below 0.05 for comparison between Fx and Fx+TBI.

**Figure 2:**
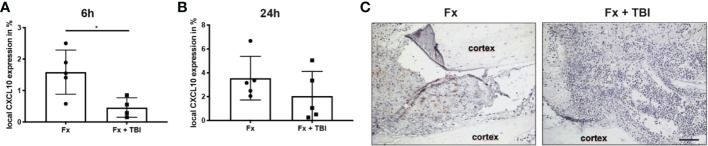
Local protein expression of CXCL10 in the hematoma. Longitudinal sections of the fracture bones were cut and stained for CXCL10. Staining was quantified by positive pixel amount relative to the total pixel. **(A)** Local CXCL10 protein expression at 6h and **(B)** 24h after fracture. **(C)** Representative images from the fracture area at 6h after fracture. Scale bar = 50 µm. * indicates p-value below 0.05 for comparison between Fx and Fx+TBI.

To analyze local immune reaction after fracture or combined trauma, several pro- and anti-inflammatory mediators known to be present in the early fracture hematoma, were determined by qPCR analysis after 6h and 24h (**Figure 1**). Interleukin-6 gene expression was significantly reduced in the hematoma of Fx+TBI mice compared to FX mice 6h, but not 24h after injury (**Figures 1A, B**). CXCL10 gene expression was significantly reduced at both time points (**Figures 1C, D**). Gene expression levels of IL-1beta, TNFalpha and IL-10 did not differ locally in the fracture hematoma at all time points (**Figures 1E–K**). To further verify the reduced expression of CXCL10 also on protein levels, immunohistochemical staining was performed (**Figure 2**). Indeed, CXCL10 was less expressed in the early fracture hematoma of Fx+TBI mice compared to Fx only mice. Expression was found in bone marrow/hematoma areas around the fracture site.

### 3.3 Immune cell infiltration into the fracture hematoma

**Figure 3:**
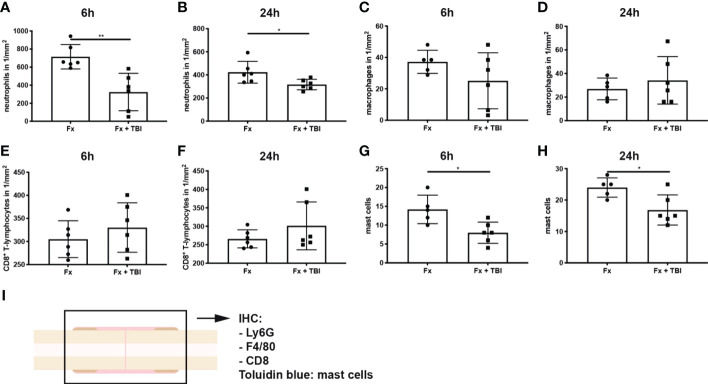
Immune cell populations in the fracture hematoma. Longitudinal sections of the fracture bones were cut and stained for immune cell markers. **(A)** Ly6G^+^ neutrophil numbers at 6h and **(B)** 24h after injury. **(C)** F4/80^+^ macrophage numbers at 6h and **(D)** 24h after injury. **(E)** CD8^+^ T-lymphocyte numbers at 6h and **(F)** 24h after injury. **(G)** Mast cell numbers were determined in Toluidin blue staining at 6h and **(H)** 24h after injury. **(I)** Experimental design. * indicates p-value below 0.05 for comparison between Fx and Fx+TBI; ** indicates p-value below 0.01 for comparison between Fx and Fx+TBI.

**Figure 4:**
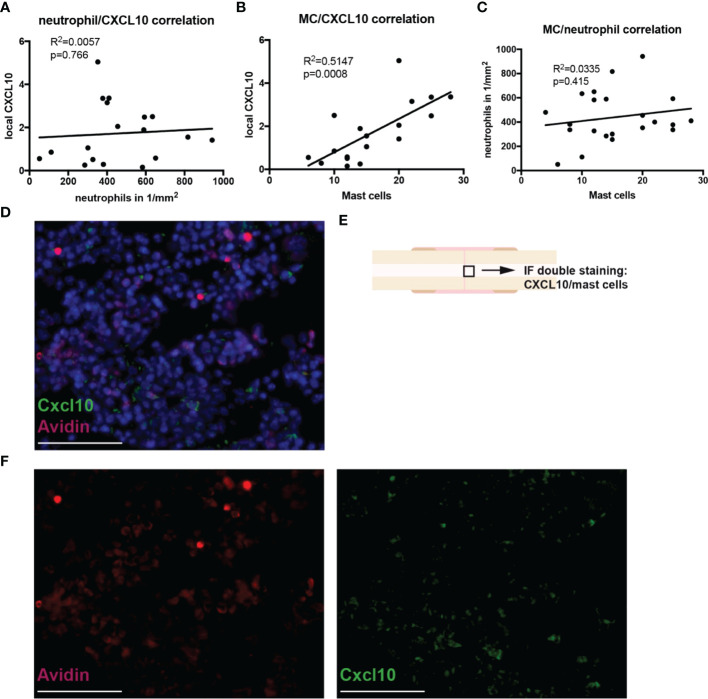
Correlations between neutrophil/mast cell numbers and CXCL10 expression in the fracture hematoma during the early inflammatory phase. Correlation analysis by simple linear regression was performed between the parameters neutrophil numbers, mast cell numbers and local CXCL10 protein expression in all samples. **(A)** Neutrophil number/CXCL10 correlation. **(B)** Mast cell number/CXCL10 correlation. **(C)** Mast cell number/neutrophil number correlation. **(D)** Immunofluoresence double staining for mast cells (Avidin staining, red) and CXCL10 (green). DNA was counterstained with Hoechst (blue). Scale bar = 50 µm. E) Black box marked the area which is shown in **(E)**. **(F)** Single fluorescent channels for Avidin (red) and CXCL10 (green). Scale bar = 50 µm.

Immune cell infiltration into the fracture hematoma was characterized by immunohistochemical staining for Ly6G (neutrophils), F4/80 (macrophages), and CD8 (cytotoxic T-lymphocytes). Mast cells were counted based on their violet-appearing granules in Toluidin blue staining. Significantly fewer neutrophils were found in the hematoma of Fx + TBI mice at both 6h and 24h after injury (**Figures 3A, B**). Macrophage and CD8^+^ T-cell numbers did not differ between the groups (**Figures 3C–F**). Further, significantly fewer mast cells were found in the hematoma of Fx + TBI mice at both 6h and 24h after injury (**Figures 3G–I**). These data indicate a dampened neutrophil and mast cell infiltration into the fracture hematoma after combined trauma. To further analyze if the reduced protein expression of CXCL10 might be due to reduced neutrophil and/or mast cell numbers, we performed a correlation analysis between the parameter’s neutrophil numbers, mast cell numbers, and local CXCL10 protein expression in all samples (**Figures 4A–C**). We detected no significant correlations between neutrophils and CXCL10 protein expression and between neutrophil and mast cell numbers, however, mast cell numbers and local CXCL10 protein expression correlated significantly with R^2 =^ 0.5417. We further established an immunofluorescence double staining method for mast cells and CXCL10 and confirmed increased CXCL10 staining in areas with many mast cells (**Figures 4D–F**). However, not all mast cells were positive for CXCL10 also non-mast cells were detected to express CXCL10, therefore we assume that mast cells are not the only cell population secreting this protein in the fracture hematoma, but might be one of the most important sources.

## 4 Discussion

Pre-clinical data strongly indicates an accelerated fracture healing after a combined traumatic brain injury (10). Clinical observations also suggested an increased callus and bone formation after combined injury (47), however strong evidence is still lacking due to the challenging monitoring of polytrauma patients (11). In preclinical models, the observation of a stronger fracture callus has been linked to altered metabolism and neuro-endocrine regulations (13, 14). In more detail, it has been shown that blood-brain barrier leakage after TBI leads to increased release of osteogenic factors from peripheral nerves (12). Further, the spenic pro- and anti-inflammatory response towards fracture was altered in TBI mice (13, 14). Other studies have linked increased bone content in the fracture callus after TBI with factors like SDF-1 (48), prolactin (49) and leptin (50). The latter was shown to influence metabolic parameters like insulin and posttraumatic osteocalcin secretion (13) and thereby altering osteoblast differentiation. Further, serum samples from patients with TBI were shown to accelerate osteogenic differentiation thereby indicating that systemic humoral factors might be involved (51, 52). However, the very early inflammatory phase of fracture healing after combined trauma has not been investigated in detail so far. This would be important since early inflammation has clearly been linked to fracture healing outcome. Therefore, the aim of this study was to analyze the early inflammatory reaction to fracture and fracture+TBI in a mouse model of ipsilateral polytrauma.

We found a dysregulated systemic inflammatory reaction in the combined trauma group with the pro-inflammatory cytokines KC and MCP1 being significantly increased. In contrast, the cytokine CXCL10 was significantly reduced in Fx+TBI mice both systemically and locally in the hematoma. This correlated significantly with reduced mast cell numbers in the fracture hematoma, while neutrophil numbers were also significantly decreased. KC, also known as CXCL1, has been shown to be highly expressed after a fracture event in mice (53) and is produced by a lot of different inflammatory cell types. KC is important for the recruitment of neutrophils to sites of injury. MCP1, also known as CCL2, is a major regulator of monocyte recruitment and is secreted by a variety of different cell types upon inflammatory stimulus (54). CXCL10, also known as IP-10, is a pro-inflammatory cytokine which is produced by mast cells, but also by some other cell types like fibroblasts and endothelial cells. Interestingly, it has been shown in the context of fracture healing, that mast cell-derived CXCL10 contributes to increased osteoclastogenesis in the fracture callus in osteoporotic mice after femur fracture (25). Mast cell-deficient mice displayed less osteoclasts in the fracture callus, a reduced callus remodelling (24) and were protected from delayed fracture healing after ovariectomy (25) and additional thoracic trauma (44). This indicates a critical role of mast cells during fracture healing and might suggest that reduced mast cell numbers could have positive effects on the healing process regarding callus bone mass, although of course callus remodeling during later healing phases is also important for fracture healing outcome in patients. We suggest that reduced mast cell numbers in the fracture hematoma and decreased local CXCL10 expression in fracture+TBI mice might lead to reduced osteoclast numbers in the later fracture callus and thereby contributing to the increase bone mass seen frequently in fracture+TBI mice in previous studies (13, 14). Clinical data supporting the hypothesis of reduced callus remodeling after TBI is available from Andermahr et al., showing reduced markers of collagen degradation in polytrauma patients with TBI (55). Further, mast cells can also influence osteoblast differentiation by secreting factors like IL-6 or Midkine (56). However, to really prove the involvement of mast cells, it would be necessary to investigate fracture healing after TBI in mast cell-deficient mice and to analyze later healing stages. This would be an interesting perspective for future studies to link mast cell appearance with osteoclastogenesis and osteoblastogenesis in the fracture callus after additional TBI.

It was also shown previously that mast cells regulate the recruitment of neutrophils to the fracture hematoma (24, 25). Therefore, it was not surprising to us that we found both reduced mast cell and neutrophil numbers in the fracture hematoma. However, we did not detect a direct correlation between mast cell and neutrophil numbers in the fracture hematoma of all animals, indicating that mast cells are not the only important regulator in neutrophil recruitment. It was also demonstrated that mast cells might be involved into the recruitment of T lymphocytes during inflammatory conditions (41). Various MC-derived chemokines and leukotrienes contribute to T cell recruitment in distinct inflammatory scenarios (39, 41, 42). Since we did not detect a difference in CD8^+^ T cells in the fracture hematoma, we suggest that during fracture healing, other stimuli are more important to recruit cells of the adaptive immune system to the fracture hematoma. This is also in line with previous data showing no differences in T cells numbers between mast cell-competent and mast cell deficient mice after fracture (24). Interestingly, previous studies have demonstrated reduced macrophage and monocyte numbers in the fracture callus of mast cell-deficient mice (24). Since we did not detect such differences in the present study, mast cells might not be critical for macrophage recruitment in the context of an additional TBI.

Limitations of our study are, as mentioned above, that we did not investigate later time points of fracture healing in this study. However, as we have demonstrated previously in that model, mice with a tibia fracture and an additional TBI displayed increased bone area in the fracture callus, while total callus area, cartilage and vascular tissue area were not altered (14). This indicates accelerated fracture healing in those mice. Another limitation is that we used a non-stabilized tibia fracture model and therefore the interfragmentary strains might differ between different animals. And since it was shown that local strains and stresses do also influence inflammation (57–59), this undefined mechanical situation might lead to higher standard deviations in the inflammatory parameters as seen in some of our datasets. Therefore, although we did a power analysis previous to our study, it would be recommended to increase sample size for future studies. Another limitation is that we did not investigate molecular mechanisms leading to the altered inflammatory status in the mice with fracture + TBI in detail. We hypothesize that traumatic brain injury leads to a recruitment of inflammatory cells to the brain rather than to the fracture location and therefore the additional injury dampens the inflammation in the fracture hematoma. There is evidence from the literature that TBI leads to influx of immune cells due to disruption of the blood-brain barrier and that this neuroinflammation can also cause long-lasting brain disfunctions (60, 61). Mast cells in the brain seems to also play a role during that process (62–64). Therefore, in our next study using the present model of fracture + TBI, we will carefully investigate also the brain tissue to further analyze neuroinflammation and bone-brain trauma crosstalk in more detail.

In conclusion, we found a dysregulated systemic inflammatory response towards fracture in mice with an additional TBI with some inflammatory cytokines being increased and some being decreased. Further, we could link decreased local expression of CXCL10 to reduced mast cell numbers in the fracture hematoma of combined trauma mice. This might contribute to accelerated fracture healing frequently seen in mice with fracture and an additional TBI as increased mast cell numbers has been linked to delayed fracture healing. Investigating the molecular mechanisms in more detail might give further insights into the molecular and cellular regulation of successful bone regeneration.

## Data availability statement

The raw data supporting the conclusions of this article will be made available by the authors, without undue reservation.

## Ethics statement

The animal study was reviewed and approved by IACUC UCSF AN143402-03B.

## Author contributions

Design of experiments: MH-L, BW, MK, KM, RM, and TM. Funding of experiments: MH-L, BW, MK, RM, AI, and TM. Conduction of experiments: MH-L, BW, KM, IL, VF, and CB. Drafting the manuscript: MH-L, RM, and TM. Editing the manuscript: MH-L, BW, KM, IL, VF, CB, AI, MK, RM, and TM. All authors contributed to the article and approved the submitted version.
